# A study of the impact of risk perception on the pro-environmental behaviour of herders in the Sanjiangyuan region

**DOI:** 10.1038/s41598-024-57336-z

**Published:** 2024-03-21

**Authors:** Zongli Zhang, Lingshu Zhang, Jina Cui

**Affiliations:** College of Finance and Economics, Qinghai University, Xining, China

**Keywords:** Environmental social sciences, Environmental economics, Psychology and behaviour

## Abstract

This study aims to study the pro-environmental behaviour of herders in the Sanjiangyuan region, a significant ecological security barrier. This paper selected 212 herding households in the Sanjiangyuan area as research subjects by random sampling method. By establishing a multivariate ordered logistics model to study the impact of risk perception on herding households' pro-environmental behaviour and introducing capital endowment as a moderating variable to analyse the moderating effect of capital endowment on the relationship of herding households' risk perception—pro-environmental behaviour. The study results show that herders's risk perception significantly affects their pro-environmental behaviour, in which environmental risk perception, economic risk perception and disease risk perception positively affect their pro-environmental behaviour. Capital endowment has a moderating role in the relationship between risk perception and the pro-environmental behaviour of herding households. Accordingly, this paper proposes to strengthen publicity and education, encourage herders to join cooperatives, and improve the ability of risk perception and other countermeasures.

## Introduction

With the rapid development of social and economic growth, the consumption demand of residents for livestock products is increasing. The livestock industry has been effectively developed and is gradually moving in an intensive and large-scale direction^1^. Animal husbandry is the pillar industry in the Sanjiangyuan area^2^, and its ecological status cannot be ignored. Accompanied by the rapid development of animal husbandry, coupled with natural factors and human activities, the ecological environment of the Sanjiangyuan region has also transformed; pasture degradation, desertification speed up, and overloading grazing not only lead to the gradual deterioration of pasture production capacity but also exacerbate the degree of desertification of the grassland, the environmental risk problems continue to come to the fore. This not only seriously impacts grassland ecology but also endangers the health of herders and even restricts the sustainable development of animal husbandry. The ecological crisis promotes integrating green agricultural activities, a critical factor in achieving socio-economic development and environmental sustainability^3^. As the direct participants and beneficiaries of grassland production and life and the ultimate implementers of pro-environmental behaviours, the physical health of herders must be protected from diseases^4^. How to fully mobilise the enthusiasm for pro-environmental behaviours of herders has become an urgent and realistic problem to be solved.

Preventing environmental pollution, protecting the ecological environment, and encouraging and promoting the pro-environmental behaviour of herders have become urgent and essential matters. In this regard, China has introduced a series of measures to support and guarantee the implementation of the "Opinions on Promoting the High-Quality Development of Livestock Husbandry" issued and implemented in September 2020, the resourceful use of livestock and poultry breeding waste, grassland ecological restoration, and to enhance the production capacity of grassland, etc., puts forward new The "Opinions on Strengthening Grassland Protection and Restoration" issued by the General Office of the State Council in 2021 has deepened the importance of grassland ecological restoration, rational use and green development. The implementation of pro-environmental behaviours cannot be separated from the extensive participation of herders, especially in the Sanjiangyuan region; the harmless treatment of sick and dead livestock, the resourceful use of manure and waste, and the standardised construction of pastureland are essential manifestations of pro-environmental behaviours. Therefore, it is significant to clarify the influencing factors of pro-environmental behaviours of herding households to alleviate the ecological and environmental problems in the Sanjiangyuan region.

In this paper, we will study the pro-environmental behaviours of herding households from three dimensions: environment, economy and disease risk. We will also consider the comprehensive influence of risk perception on pro-environmental behaviours. The paper takes the herding households in the Sanjiangyuan region as the research object, explores the impact of risk perception on pro-environmental behaviours, and further explores the effect of capital endowment on risk perception and pro-environmental behaviours, which can help to broaden the connotation of pro-environmental behaviours, clarify the influence of various types of risk perception on the pro-environmental behaviours of the herding households, and provide a reference for the implementation of pro-environmental behaviours of herding households in other regions, which is of great significance for the realisation of sustainable development of the Sanjiangyuan region. It is of great importance to the sustainable development of the Sanjiangyuan region.

## Literature review

Risk perception and pro-environmental behaviours and awareness of these issues increase the importance of our understanding of ecological degradation and its elements; this study investigates the impact of risk perception on the pro-environmental behaviours of pastoral households in the Sanjiangyuan region; we divide the literature into two parts to illustrate the relationship between the research variables. The first part describes the relationship between risk perception and pro-environmental behaviour of herder households in the Sanjiangyuan region, and the second part discusses the relationship between capital endowment in risk perception and pro-environmental behaviour of herder households.

### Impact of risk perception on herders' pro-environmental behaviour

Research on risk perception dates back to the 1940s when Slovic defined risk perception as an individual's overall understanding and perception of the objective world as a result of subjective feelings and experiences^5^. Behavioural economics theory suggests that psychological factors should be introduced to analyse human decision-making behaviour effectively^6^. Risk is ubiquitous and can be divided into many categories of risk according to different classifications, such as natural risk and social risk according to the source, life risk, ecological risk and economic risk according to the consequences^7^. Liu Li et al. found that risk perception positively affects farmers' adoption of soil and water conservation farming techniques^8^. Du Sanxia et al. pointed out that the stronger the risk perception, the less likely farmers are to adopt biopesticide technology^9^. Liu Zheng et al. found that environmental risk perception was the main factor influencing farmers' pro-environmental behaviour^10^. Guo Qinghui et al. showed that farmers' perception of ecological pollution positively influenced their pro-environmental behaviour^11^. Gaihao et al. found that perceived value and government regulation influence farmers' mechanised straw return behaviour and can reduce environmental pollution^12^. Wu Linhai et al. found that pig epidemics, perceptions of epidemic prevention, and years of farming experience affect the behaviour of sick and dead pig disposal^13^. Sun Yu and other scholars found that risk perception negatively affects behaviour through willingness^14^. Wang Qian et al. pointed out that risk perception significantly negatively should affect agricultural land transfer behaviour^15^. Li et al. found that environmental risk perception significantly negatively affects green production behaviour^16^. Zhu Hui found that ecological pollution risk perception does not significantly affect pro-environmental behaviour^17^. Combing through the literature, it is found that the study of the effect of risk perception on behaviour has not yet formed a unified conclusion. Therefore, it is of great theoretical significance to explore the impact of risk perception on pro-environmental behaviour.

Based on the risk aversion theory, individuals will avoid risks and reduce losses when making behavioural decisions, in line with the rational economic man assumption, specific to the implementation of pro-environmental behaviours of herdsmen, herdsmen will be a comprehensive measure of the external environment, based on their own experience, the external information through the screening of the complete trade-offs, to make a response to the external impacts and provide the basis for the behavioural decisions of avoidance, change, and acceptance of risk^18^. Scholars have conducted many studies on the effects of risk perception on behaviour. Still, most started from a single risk and explored the comprehensive effect of multiple risks less. The research on the impact mechanism of risk perception on pro-environmental behaviours needs to be discussed in depth. Combining with the actual situation of the Sanjiangyuan region, this paper comprehensively researches the pastoral household's risk perception from the three dimensions of the perception of environmental risk, economic risk, and disease risk. Based on the above analyses, this paper puts forward the following hypotheses:

**H1**: Risk perception positively influences the pro-environmental behaviour of herding households in the Sanjiangyuan region.

**H1a**: Environmental risk perception positively influences the pro-environmental behaviour of herding households in the Sanjiangyuan region.

**H1b**: Perceived economic risk positively influences the pro-environmental behaviour of herding households in Sanjiangyuan.

**H1c**: Disease risk perception positively affects the pro-environmental behaviours of herding households in Sanjiangyuan.

### Moderating effect of capital endowment on the relationship between risk perception and pro-environmental behaviour of pastoral households

Capital endowment is the resources and capabilities available to herdsmen in production and life. The more common classifications of capital endowment are natural capital, human capital, physical capital, financial capital and social capital^19^. Capital endowment, as an essential resource, is the material basis and guarantee for the survival and development of the herdsmen of the Sanjiangyuan, and the research has shown that the amount of livelihood capital will directly affect the herdsmen's willingness to protect the grassland^20^. Livelihood capital not only affects the quality of life of herding households but also influences the decision-making of herding households' production behaviour. It has been found that livelihood capital is a significant factor affecting farmers' risk-coping strategies^21^, in which social capital not only significantly affects farmers' perception^22^ but also effectively mitigates the impacts of risks and provides risk protection for farmers^23^. Subject to the constraints of many real-life factors, even if pastoralists have a high-risk perception of the external environment, it is often difficult to turn into practical action to avoid risk exploring the relationship between capital endowment and pastoralists' risk perception has a significant impact on the implementation of pro-environmental behaviour of pastoralists. As a labour-intensive industry, animal husbandry is very dependent on human capital, and it is necessary to ensure the sustainability of human capital and the quality of its health. Herding households with a high proportion of animal husbandry labour will engage in non-animal husbandry industries to obtain income while satisfying the needs of animal husbandry production, and the abundance of human capital not only affects herding household's livelihood decision-making but also influences herding household's perception of risk; physical capital is the basis and prerequisite for herding households to maintain their daily livelihoods. Physical capital is the foundation and prerequisite for herding households to sustain their daily livelihoods; the higher the level of physical capital, the more energy herding households have to pay attention to the ecological environment; social capital significantly and positively affects the quality of life^24^, in addition to the fact that social capital can help to herd households to obtain more information and external resources, and herding households with a higher level of social capital are more likely to get valuable information. Based on the above analyses, this paper proposes the hypothesis:

**H2**: There is a positive moderating effect of capital endowment on the relationship between risk perception and pro-environmental behaviour of pastoral households.

**H2a**: Human capital positively moderates the relationship between perceived risk and pro-environmental behaviour of herding households.

**H2b**: Physical capital positively moderates the relationship between perceived risk and pro-environmental behaviour of herding households.

**H2c**: Social capital positively moderates the relationship between herding households' risk perception and pro-environmental behaviour.

## Methods

### Data sources and sample characteristics

The data used in this paper come from the research conducted in April–May 2022 in the Sanjiangyuan region by a research group from Qinghai University. The study used random sampling to determine the research subjects. To improve the effectiveness and accuracy of the research, the group provided systematic training and several question-and-answer sessions to the participants before the field research and conducted a pre-survey of the questionnaire, which was further modified according to the results of the pre-survey to provide a basis for the following work. A total of 241 questionnaires were distributed, and 212 were recovered, with a validity rate of 87.96%.

*Statements*: All methods were performed in accordance with relevant guidelines and regulations, and all experimental protocols were approved by the Department of Tourism and Business Administration, College of Finance and Economics, Qinghai University. Informed consent was obtained from all the participants or their legal guardians.

Table 1 lists the definitions and characteristics of the sample data. From the essential elements of the sample, the age distribution of the interviewees is mainly 60 years old and below, accounting for 99.06% of the total sample ratio; among the sample families, the number of family labourers is mainly in herding families with 3–5 persons; and the annual income from family herding and farming is primarily in the range of RMB 10,000–50,000yuan, accounting for 45.28% of the total sample ratio. Thus, the sample households are representative to a certain extent.

**Table 1:** Basic characteristics of the sample.

Variables	Definition and assignment	No. of samples/pcs	Proportion/per cent	Variables	Definition and assignment	No. of samples/pcs	Proportion/per cent
Genders	Male	100	47.17	Number of family labourers	≤ 2	82	38.68
	Female	112	52.83		[3, 5]	119	56.13
Age/years	≤ 30	129	60.85		[6, 8]	9	4.25
	[31, 45]	46	21.70		> 8	2	0.94
	[46, 60]	35	16.51	Grassland area/acre	≤ 50	38	17.92
	≥ 61	2	0.94		[51, 150]	83	39.15
Educational level	Illiterate	43	20.28		[151, 250]	51	24.06
	Primary school	52	24.53		≥ 251	40	18.87
	Junior high School	41	19.34	Number of pasture blocks	≤ 2	68	32.07
	Senior high school	23	10.85		[3, 5]	111	52.36
	College and above	53	25.00		[6, 8]	28	13.21
The presence of village cadres among family members	Yes	56	26.42		> 8	5	2.36
	No	156	73.58	Annual income from family pastoral farming/$10,000	≤ 1	78	36.79
Type of part-time employment	Pure Livestock Husbandry	85	40.09		(1, 5]	96	45.28
	Livestock part-time	105	49.53		(5, 10]	33	15.57
	Labour and business	22	10.38		> 10	5	2.36

### Methodology

#### Multivariate ordered logistic models

The explanatory variable in this paper is the pro-environmental behaviour of herdsmen P, which belongs to the ordered discrete variables, in which the value P = P(y = 1) = F(βi Xi) is assigned to each level P, where (i = 1, 2, 3, 4, 5), y = 1 means that the pro-environmental behaviour is the weakest, y = 2 means that the pro-environmental behaviour is more vulnerable, and so on, y = 5 means that the pro-environmental behaviour is the strongest. Xi means the ith factor that affects the pro-environmental behaviour of the herders. Environmental behaviour. Therefore, a multivariate ordered logistic model was established:

1 $${\text{ln}}\left[\frac{\sum_{i=1}^{j}{P}_{i}}{1|\sum_{i=1}^{j}{P}_{i}}\right]={a}_{j}+\sum_{i=1}^{m}{\beta }_{i}{x}_{i}$$

Pj represents the probability of a given level of pro-environmental behaviour, αi is the model intercept, and βi is the partial regression coefficient.

#### Moderated effects model

The moderated effects model is set up as follows

2 $${Y}_{i}={\beta }_{0}+{\beta }_{1}{X}_{i}+\underset{i=2}{\sum^{n}}{\beta }_{i}{Z}_{i}+{\varepsilon }_{i}$$

3 $${Y}_{i}={\beta }_{0}+{\beta }_{1}{X}_{i}+{\beta }_{2}{M}_{i}+{\beta }_{3}{X}_{i}{M}_{i}+\underset{i=4}{\sum^{n}}{\beta }_{i}{Z}_{i}+{\varepsilon }_{i}$$

In Eqs. (2) and (3), X is the core explanatory variable risk perception, which represents environmental risk perception/economic risk perception/disease risk perception, Z means the group of control variables affecting the pro-environmental behaviours of herders, ε defines the random error term, M is the capital endowment, ${X}_{i}{M}_{i}$ is the interaction term of the explanatory variables and the moderating variables, and ${\beta }_{0}$, ${\beta }_{1}$,${\beta }_{2}$,${\beta }_{3}$ are the coefficients, and the regression coefficients can be determined by examining whether or not there is a moderating effect of ${X}_{i}{M}_{i}$ regression coefficients can determine whether capital endowment has a moderating role in the relationship between risk perception and the implementation of pro-environmental behaviours by herders. If the regression coefficient of ${X}_{i}{M}_{i}$ is significant, it means that there is a moderating effect.

### Description of variables

#### Explained variables

The explanatory variables of this paper are the pro-environmental behaviours of herding households. Combining the actual situation of the Sanjiangyuan region, this paper measures the pro-environmental behaviours of flocking households from standardised construction, harmless treatment, resource utilisation, standardised herding, and hygiene and epidemic prevention.

#### Core explanatory variables

This study focuses on Sanjiangyuan herding households' risks in production and life. It measures the risk perception of Sanjiangyuan flocking households through three dimensions: environment, economy and disease. Perceived environmental risks were measured by three questions: "the impact of faeces and waste on the ecological environment", "the degree of pasture degradation", and "the severity of natural disasters (e.g., snowstorms)". The perception of economic risk was measured by asking questions about pasture degradation and the severity of natural disasters. Economic risk perception was measured by asking the herders about the "cost of pasture management", "economic loss caused by natural disasters (e.g., snowstorms)", and "reduction in the number of herded animals". Disease risk perception was measured by "immediate harmless disposal of sick and dead animals", "regular disinfection and sterilisation of feed, water and dung channels", and "familiarity with disease prevention measures". The three measures are "familiarity with disease prevention measures".

#### Moderating variables

The moderating variable is capital endowment, and this paper mainly measures the capital endowment of herding households from three aspects: human capital, physical capital and social capital. Human capital includes explicitly the labour and health status of livestock farming and the frequency of livestock farming skills training for herders; physical capital refers to the essential means of production that support herders in livestock farming; and social capital refers to the social resources that herders can use.

#### Control variables

It has been shown in the literature that the characteristics of an individual can have an impact on the results of the study^25–27^, such as age^28^, education level^29^, and income^30,31^can have an impact on the adoption behaviour of an individual. Therefore, in this paper, gender, age, presence of village cadres in the family members, and education level of the respondents were set as control variables during the study. The variables and descriptive statistical characteristics are shown in Table 2.

**Table 2:** Variable selection and descriptive statistics.

Variable type	Variable name	Average value	Standard deviation
Explanatory variables	Perceived Environmental Risk	2.830	0.546
	Economy Risk perception	2.917	0.477
	Disease Risk perception	3.151	0.483
Moderating variables	Human capital	2.976	0.575
	Physical capital	2.085	0.648
	Social capital	3.299	0.601
Control variables	Age	1.575	0.796
	Gender	1.528	0.500
	The presence of village cadres among family members	1.736	0.442
	Educational level	2.958	1.474

## Results

### An empirical analysis of the impact of risk perception on the pro-environmental behaviour of herding households in the Sanjiangyuan region

A reliability test was conducted to ensure the reliability and validity of the questionnaire. Its Cronbach reliability coefficients were all above 0.7, which indicates that the internal consistency is better. In this paper, in analysing the respective variables through the multicollinearity judgement method, the maximum value of its variance inflation factor was 1.523, indicating no multicollinearity between the independent variables. According to the micro-data obtained from the research, the influence of herders’ risk perception on pro-environmental behaviour was analysed by SPSS27.0 statistical software, and the specific regression results are shown in Tables 3 and 4.

**Table 3:** Results of regression analysis of total indicators of risk perception on pro-environmental behaviours of pastoral households in the Sanjiangyuan region.

Variable type	Variable name	B-value	Standardised regression coefficient	T-value	Sig
Explanatory Variables	Risk perception	2.670***	0.470	3.031	0.002
	Perceived Environmental Risk	0.461***	0.139	2.605	0.008
	Economy Risk perception	0.334**	0.088	2.012	0.044
	Disease Risk perception	0.368**	0.098	2.091	0.037
Other variables	Controlled				

***Significant value at 1%, **significant value at 5%, *significant value at 10%.

**Table 4:** Results of regression analysis of the impact of risk perception sub-indicators on the pro-environmental behaviour of herding households in the Sanjiangyuan region.

Variable type	Variable name	Variable definitions	B-value	Standardised regression coefficient	Sig
Explanatory Variables	Perceived Environmental Risk	Ecological impacts of faeces and wastes	0.423**	0.072	0.010
		Degree of pasture degradation	0.378**	0.075	0.019
		Severity of natural disasters (e.g., snowstorms)	0.225*	0.072	0.096
	Economy Risk perception	Costs of pasture management	0.118	0.077	0.456
		Economic losses due to natural disasters (e.g. snowstorms)	0.246*	0.076	0.086
		Reduction in the number of stocked animals	0.099	0.072	0.551
	Disease Risk perception	Immediate harmless treatment of sick and dead livestock	0.391***	0.075	0.008
		Regular disinfection and sterilisation of feed, water and manure channels	0.412***	0.080	0.009
		Familiarity with disease prevention measures	0.160	0.076	0.335
Control Variables	Age		0.515**	0.088	0.010
	Gender		0.003	0.075	0.992
	Educational level		− 0.069	0.082	0.497
	The presence of village cadres among family members		0.595*	0.075	0.053

***Significant value at 1%, **significant value at 5%, *significant value at 10%.

As seen from Table 3, the risk perception of herding households passed the 1% positive significance test, which verified hypothesis H1 of this paper. The effects of risk perception sub-dimensions on the pro-environmental behaviours of herding households in the Sanjiangyuan region all passed the 5% positive significance test. The hypotheses H1a, H1b and H1c were further verified. Accordingly, this paper further explores the influence of risk perception sub-indicators on pro-environmental behaviour, and the specific regression results are shown in Table 4.

Table 4 demonstrates the following regression results: the impact of faeces and waste on the ecological environment, pasture degradation, and the severity of natural disasters (e.g., snowstorms) are positively significant at the 5%, 5%, and 10% levels, respectively. Herders know that dung and grazing waste affect the environment and further aggravate pasture degradation. In contrast, herders are more impressed by the hazards caused by natural disasters. Implementing pro-environmental behaviours can mitigate pasture degradation and protect the environment; thus, herders will tend to implement pro-environmental behaviours.

The impact of economic risk perception on pro-environmental behaviour showed that economic losses caused by natural disasters (e.g., snowstorms) passed the 10% positive significance test. In contrast, other aspects of economic risk perception failed the significance test. On the one hand, this is because economic losses are directly related to herders' income and are perceived more strongly; on the other hand, because herders have a reverence for nature, they follow the laws of nature and emphasise self-repairing of the pasture, and livestock is the primary source of income for herders, which influences and determines herders' livelihoods so that other aspects of the perception of economic risk are not significant.

The effect of disease risk perception on pro-environmental behaviours showed that immediate harmless disposal of sick and dead livestock and regular disinfection and sterilisation of feed, water and dung channels were positively significant at the 1% level. At the same time, familiarity with epidemic preventive measures did not pass the significance test. This may be because immediate disposal of sick and dead livestock and regular disinfection and sterilisation can essentially block the spread of the disease. In contrast, the suddenness of livestock epidemics makes it more difficult for herders to be aware of them and, therefore, do not implement pro-environmental behaviours promptly.

### The impact of personal characteristics on the pro-environmental behaviour of herders

Age and the presence or absence of village cadres in the household were all positively significant at the 10% level, while gender and education failed the significance test. This may be due to the low level of education of the herders and the fact that older herders and herder families with village cadres in the household are the ones who call for pro-environmental behaviours and propagate them, thus encouraging herders to practice pro-environmental behaviours.

### The moderating effect of capital endowment in the relationship between risk perception and pro-environmental behaviour of herding households

This paper analyses the effects of total capital endowment indicators and sub-indicators on risk perception and pro-environmental behaviours of pastoral households in the Sanjiangyuan region, and the specific regression results are shown in Table 5.

**Table 5:** The aggregate moderating effect of capital endowment on the relationship between risk perception and pro-environmental behaviour of herding households.

	Capital endowment	
	Ratio	Standard error
Risk perception	0.172***	0.183
Other variables	Controlled	

***Significant value at 1%, **significant value at 5%, *significant value at 10%.

As seen from Table 5, the effect of risk perception on the pro-environmental behaviour of herding households under the moderating impact of capital endowment passes the 1% positive significance test, which verifies the hypothesis of this paper, H2. Accordingly, this paper further explores the regression results of the dimensions of capital endowment on the risk perception and the pro-environmental behaviour of the herding households in the analysis of the regression results of each of the dimensions of capital endowment from the three dimensions of the perception of risk, as shown in Tables 6, 7, and 8.

**Table 6:** The moderating effect of human capital on the relationship between risk perception and pro-environmental behaviour of herders.

	Human capital (HC)	Perceived environmental risk (PER)	Economy risk perception (ERP)	Disease risk perception (DRP)
HC	0.178*** (0.137)	0.247*** (0.137)	0.164** (0.164)	0.261*** (0.156)
Age	0.230*** (0.110)	0.243*** (0.108)	0.236*** (0.113)	0.238*** (0.110)
Gender	− 0.030 (0.157)	− 0.013 (0.154)	0.018 (0.160)	0.022 (0.157)
The presence of village cadres among family members	0.109* (0.172)	0.108* (0.170)	0.123* (0.177)	0.088 (0.181)
Educational level	− 0.013 (0.058)	− 0.048 (0.060)	− 0.086 (0.062)	− 0.069 (0.059)
PER		0.284*** (0.136)		
HC*PER		0.151** (0.217)		
ERP			0.191*** (0.162)	
HC*ERP			0.133* (0.266)	
DRP				0.266*** (0.156)
HC*DRP				0.138** (0.249)

***Significant value at 1%, **significant value at 5%, *significant value at 10%. Standard errors in parentheses.

**Table 7:** Moderating effect of physical capital on the relationship between risk perception and pro-environmental behaviour of herders.

	Physical capital (PC)	Perceived environmental risk (PER)	Economy risk perception (ERP)	Disease risk perception (DRP)
PC	0.127* (0.123)	0.273*** (0.136)	0.191*** (0.162)	0.282*** (0.155)
Other variables	Omitted			
PC*PER		0.119* (0.207)		
PC*ERP			0.191*** (0.162)	
PC*DRP				− 0.042 (0.225)

The estimation results of other variables are similar to the above table, omitted here to reduce redundancy, as below.

**Table 8:** Moderating effect of social capital on the relationship between risk perception and pro-environmental behaviour of herders.

	Social capital	Perceived environmental risk (PER)	Economy risk perception (ERP)	Disease risk perception (DRP)
SC	0.025 (0.136)	0.269*** (0.138)	0.189*** (0.163)	0.266*** (0.159)
Other variables	Omitted			
SC*PER		0.145** (0.214)		
SC*ERP			0.133* (0.233)	
SC*DRP				0.092 (0.253)

As shown in Table 6, under the moderating effect of human capital, environmental risk perception, economic risk perception, and disease risk perception are positively significant at the 5%, 10%, and 5% levels, respectively, which may be because pastoral households with abundant human capital can learn and master new animal husbandry skills, have a higher perception of wind, and can implement pro-environmental behaviours in proactive response to risk shocks, verifying the hypothesis H2a of this paper.

As shown in Table 7, under the moderating effect of physical capital, environmental and economic risk perceptions are positively significant at the 10% and 1% levels, respectively. In contrast, disease risk perception does not pass the moderating test, which may be because when herders perceive risks, physical capital can mitigate the risk shocks and motivate them to implement pro-environmental behaviours, which verifies hypothesis H2b of this paper.

As can be seen from Table 8, under the moderating effect of social capital, the EEP risk perception and economic risk perception are positively significant at the 5% and 10% levels, respectively, which may be because long-term social interactions will strengthen the environmental cognition of herders, which in turn will prompt them to implement pro-environmental behaviours actively, verifying the hypothesis H2c of this paper.

## Discussion

As direct participants and beneficiaries of grassland production and life, herders are the ultimate implementers of pro-environmental behaviours. However, due to multiple factors such as herders' level and family economic status, there are differences in the acceptance and adoption of pro-environmental behaviours by herders, so how to break through the constraints and promote the implementation of pro-environmental behaviours by herders is of great significance for the ecological and economic social development of the Three Rivers source area. Therefore, it is of practical value to study the effect of risk perception on pro-environmental behaviours of herding households and to formulate targeted policies to motivate them to implement pro-environmental behaviours. This paper puts the relationship between capital endowment, risk perception and pro-environmental behaviour into the same framework system. It analyses the impact of risk perception on the pro-environmental behaviour of pastoral households in the Sanjiangyuan rural area under the moderating effect of capital endowment.

### How does risk perception affect pro-environmental behaviour?

Zhang Yu's study found that farmers' environmental risk perception positively affects the implementation of their ecological behaviours^7^; Fan Wenjie and Cui Jina's empirical study found that herders' risk perception significantly positively affects pro-environmental behaviours^32^. In this study, the impact of herders' risk perception on pro-environmental behaviours passed the 1% positive significance test, indicating that herders' risk perception positively affects pro-environmental behaviours; the effect of risk perception sub-dimensions on pro-environmental behaviours passed the 5% positive significance test, meaning that different dimensions of risk perception play a positive and significant role in affecting pro-environmental behaviours. Research hypotheses H1, H1a, H1b, and H1c were proved and were consistent with the findings of the above scholars. In addition, this study found that the effect of environmental risk perception on pro-environmental behaviours of herder households was more significant. Herding households can realise that dung and waste produced by grazing will affect the environment and further aggravate pasture degradation. In contrast, herding households have a more profound impression of the hazards caused by natural disasters. Implementing pro-environmental behaviours has the effect of mitigating pasture degradation and protecting the environment so that the impact of environmental risk perception on pro-environmental behaviours is more significant.

### How does capital endowment regulate the relationship between herders' risk perception and pro-environmental behaviour?

In the research on the effect of capital endowment, Wang Yuanyu believes that human capital and social capital have a positive impact on pig farmers' intensive production behaviour^33^; Geng Strait believes that human capital and social capital play a positive effect on farmers' green production behaviour, while physical capital has a significant inhibitory effect on farmers' green production behaviour^34^. In this study, the impact of risk perception on herdsmen's pro-environmental behaviour under the moderating effect of capital endowment passed the 1% positive significance test, indicating that capital endowment positively moderates the relationship between herdsmen's risk perception and pro-environmental behaviour. Meanwhile, in terms of dimensions, human capital, physical capital, and social capital have positive moderating effects on the relationship between risk perception and the pro-environmental behaviour of herding households. The research hypotheses H2, H2a, H2b, and H2c were all confirmed and corroborated with the results of the above scholars' studies. It is also worth noting that physical capital did not play a significant role in moderating the relationship between disease risk perception and pro-environmental behaviour among pastoralists, which is inconsistent with the expected results. The possible explanation for this is that rural households engaged in pastoral production own many agricultural machinery and tools, which are costly to acquire, use and maintain, and that financial constraints lead to a lower willingness of rural households to invest in other areas.

## Conclusions

Based on the field research data of 212 herding households in the Sanjiangyuan area, this paper incorporates risk perception, capital endowment and herding households' pro-environmental behaviour into the analytical framework, objectively analyses the impact of risk perception on herding households' pro-environmental behaviour, and further analyses the moderating effect of capital endowment on the relationship between risk perception and herding households' pro-environmental behaviour on this basis. The study results show that (1) risk perception positively affects herding households' pro-environmental behaviour. Among them, the effect of environmental risk perception on the pro-environmental behaviour of herding households is more significant. (2) Capital endowment has a positive moderating effect on the relationship between risk perception and pro-environmental behaviours of herding households, and human capital, physical capital and social capital all have a positive moderating effect on herding households' risk perception and pro-environmental behaviours.

In practical terms, these recommendations help achieve sustainable development in the Sanjiangyuan region and improve the region's environmental quality. First, the empirical results show that risk perception positively affects herder pro-environmental behaviour and that human capital, physical capital and social capital all positively regulate herder risk perception-herder pro-environmental behaviour. Therefore, we need to improve the risk perception ability, risk prevention, and control level of herders in Sanjiangyuan to reduce economic losses. In addition, we should encourage herders to join cooperatives, participate in various forms of skills training, and increase the level of trust among herders to improve their capital endowment. As we all know, as the main body of implementing pro-environmental behaviours, herders' cognition of pro-environment directly affects their pro-environmental willingness and pro-environmental behaviours; therefore, it is necessary to increase the publicity of pro-environmental behaviours, improve the level of pro-environmental cognition of herders, prompt herders to generate cheerful pro-environmental willingness and stimulate herders to implement pro-environmental behaviours positively and actively.

### Supplementary Information

Supplementary Information.

## Data Availability

The datasets generated and analysed during the current study are not publicly available due to the confidentiality of the follow-up study. Still, they are available from the corresponding author upon reasonable request.

## References

[1] Zhang L, Li H (2019). Pollution of the ecosystem in livestock production and its prevention and control. Mod. Anim. Husb. Sci. Technol..

[2] Xiong H (2022). Study on the economic development of livestock husbandry in Sanjiangyuan ecological reserve of Qinghai Province. Fortune Today.

[3] Gholamrezai S, Aliabadi V, Ataei P (2021). Understanding the pro-environmental behavior among green poultry farmers: Application of behavioral theories. Environ. Dev. Sustain..

[4] Karimi, H. & Ataei, P. Perceived social risks and farmers’ behavior in using urban wastewater in their farms. *Environ. Sustain. Indic*, 100301 (2023).

[5] Slovic P (1987). Perception of risk. Science.

[6] Pan D, Kong F (2015). Behavioural analysis of farmers' choice of environmentally friendly livestock and poultry manure treatment methods: A case study of pig farming. Chin. Rural Econ..

[7] Zhang Y (2016). An Empirical Study on the Relationship Between Environmental Risk Perception, Environmental Regulation and Environmental Behaviour.

[8] Liu L, Chu LQ, Jiang ZD (2020). Influence of technology cognition and risk perception on the willingness to adopt soil and water conservation tillage technologies and its inter-generational differences. Resour. Sci..

[9] Du S, Luo X, Huang Y, Tang L, Yu W (2021). Risk perception, agricultural social services and rice farmers' biopesticide technology adoption behaviour. Resour. Environ. Yangtze Basin.

[10] Liu Z, Zhou J (2018). Information ability, perception of environmental risk and farmers’ environmentally friendly behavior adoption—Based on empirical test of the sample of broiler farmers in Liaoning Province. J. Agrotech. Econ.

[11] Guo Q, Li H, Li S (2019). Analysis of the influence of personal norms on farmers’ pro-environmental behavior—Based on the extended theory of norm-activation. Resour. Environ. Yangtze Basin.

[12] Gai H, Yan T, Zhang J (2020). Perceived value, government regulations and farmers’ behaviors of continued mechanized operation of straw returning to the field: An analysis based on survey data from 1288 Farmersin three provinces of Hebei, Anhui and Hubei. Chin. Rural Econ..

[13] Wu LH, Xu GY, Hu WY (2015). Simulating selection process of pig farmers' disposing behaviour of dead pigs: Simulation experiment method. J. Nanjing Agric. Univ. (Soc. Sci. Ed.).

[14] Sun Y, Luo WL, Zhang C (2022). How does public perception affect willingness and behaviour to use face recognition technology? A moderated mediation model based on usage context. Public Adm. Rev..

[15] Wang Q, Guan R, Yu J (2019). Analysis of the impact of risk attitude and risk perception on farmers' farmland transfer behaviour-based on panel data of 1429 farmers in Henan, Luwan, Hebei and Suzhou. J. Huazhong Agric. Univ. (Soc. Sci. Ed.).

[16] Li MY (2020). Factors affecting the willingness of agricultural green production from the perspective of farmers' perceptions. Sci. Total Environ..

[17] Zhu H (2017). Environmental knowledge, risk perception and youth environmentally friendly behaviour. Contemp. Youth Stud..

[18] Xu C, Chen R, Jiang Z (2018). A study of external shocks, risk appetite and adoption of low carbon agricultural technologies by farmers. Sci. Technol. Manag. Res..

[19] Dfid U (1999). Sustainable Livelihoods Guidance Sheets.

[20] Zhang Y, Xu T, Zhao MJ (2019). Ecological cognition, family livelihood capital and willingness of herdsmen to participate in grassland protection. J. Arid. Land Resour. Environ.

[21] Su F, Shang H (2012). The impact of farm households' livelihood capital on their risk coping strategies: A case study of Zhangye City in the Black River Basin. Chin. Rural Econ..

[22] Deressa TT, Hassan RM, Ringler C (2011). Perception of and adaptation to climate change by farmers in the Nile basin of Ethiopia. J. Agric. Sci..

[23] Cai QH, Zhu YC (2016). Influence of social capital and income inequality on village collective action: Based on farmers’ participation in the maintenance of small irrigation systems in three provinces. J. Public Manag..

[24] Larnyo E (2021). Assessing the impact of social media use on everyday emotion in health crises: A study of international students in China during COVID-19. Healthcare.

[25] Zhang Y (2019). Public risk perception, government trust and conflict participation intentions for environmental neighbourhood facilities. Adm. Tribune.

[26] Huang J, Qi L, Chen R (2008). Knowledge of technical information, risk preferences and pesticide application by farmers. J. Manag. World.

[27] Cheng L, Zhang J, He K (2019). Analysis of the impact of network embedding and risk perception on farmers' adoption behaviour of green farming technology—Based on survey data from 615 farmers in Hubei Province. Resour. Environ. Yangtze Basin.

[28] Hou XK, Liu TJ, Huang T (2019). Adoption behavior and income effects of green agricultural technology for farmers. J. Northwest Univ. Agric. For. (Soc. Sci. Ed.).

[29] Wang P, Wang L (2022). A study of the effect of farmers' perception on the adoption behaviour of maize stover return technology in an environmental regulatory context. J. Arid Land Resour. Environ..

[30] Wu B, Guo J, Ma J (2014). Social capital and informal risk sharing mechanisms for farm households: Theoretical framework and empirical evidence. J. Agrotech. Econ..

[31] Zhang T, Yan T, He K, Zhang J (2019). Altruistic tendencies, limited rationality and farmers' green agricultural technology adoption behaviour. J. Northwest A&F Univ. (Soc. Sci. Ed.).

[32] Fan W, Cui J (2023). A study on the impact of pastoralists' risk perception on pro-environmental behaviour based on the perspective of environmental regulation—A case study of the Sanjiangyuan Region. J. Arid Land Resour. Environ..

[33] Wang Y (2023). A Study of Capital Endowment, Green Perceptions and Cleaner Production Behaviour of Pig Farmers.

[34] Geng H (2023). A Study of the Effects of Capital Endowment and Green Perception on the Green Production Behaviour of Farm Households.

