# Supplementary material for: A study of the impact of risk perception on the pro-environmental behaviour of herders in the Sanjiangyuan region
Source: Sci Rep. 2024 Mar 21;14:6788. doi: 10.1038/s41598-024-57336-z (PMC10957992; doi:10.1038/s41598-024-57336-z)
Supplement: Supplementary file 1 — Supplementary Information. [file 41598_2024_57336_MOESM1_ESM.pdf]

Questionnaire on Pro-Environmental Behaviour of Herding Households and its Influencing Factors

No.: Place of survey:

Hello! I am a member of the research group of Qinghai University. Now, we are conducting a questionnaire survey on the basic situation of pro-environmental behaviours of herding households. I want to get relevant information; thank you for assisting us with the study in your busy schedule. The questionnaire will be used only for academic research, will be kept confidential, and will not harm your interests.

Basic information

1. Age: ○18-30 years old ○31-45 years old ○46-60 years old ○60 years old and above
2. Gender: ○Male ○Female
3. Number of household laborers: ○1-2 persons ○3-5 persons ○6-8 persons ○8-10 persons ○10 or more persons

Part I

1. Are you willing to engage in environmental protection behaviour while grazing?

○Yes ○No

2. You have already carried out behaviours to protect the environment while grazing.

○Yes ○No

3. Are you willing to reduce the use of feed additives and veterinary drugs?

○Yes ○No

4、If reducing the use of feed additives and veterinary drugs will decrease your earnings, how much can you afford to lose?

○0-500 yuan ○501-1000 yuan ○1001-1500 yuan ○1501-2000 yuan ○More than 2000 yuan

5、How often do you use veterinary drugs in a year?

○No use ○1-2 times ○3-5 times ○6-8 times ○More than 8 times

6、You are under the guidance of professionals or by instructions every time you dispense veterinary drugs.

○Never ○Occasionally ○Generally ○Much ○Always

7. You will intentionally control the number of cattle and sheep grazing to maintain ecological balance.

○Never ○Occasionally ○Generally ○Much ○Always

8. The more cows and sheep in the pasture, the better your opinion on this opinion.

○Disagree ○Somewhat ○Fair ○Agree

9. How strong is your willingness to reduce feed additives and veterinary drugs in grazing?

○1 ○2 ○3 ○4 ○5 (1 indicates the weakest willingness, 5 the strongest)

10. You are willing to conduct regular disinfection, decontamination and desertification control of pastureland to construct sustainable pastureland.

○Yes ○No

11. If regular testing, disinfection, pest control, desertification control, and pasture construction on your pasture would decrease your earnings, how much can you afford to lose?

○0-500 yuan ○501-1000 yuan ○1001-1500 yuan ○1501-2000 yuan ○More than 2000 yuan

12. Is your farm a quality farm recognised by the government or a co-operative?

○Yes ○No

13、You have adopted the grazing methods of grass-based grazing, grass-based animal husbandry, and rotational grazing of pasture

○Yes ○No

14. How much effort have you made to prevent rodent infestation and desertification on your ranch?

○0% ○25% ○50% ○75% ○100%

15、How do you store your veterinary medicines?

○ in the pasture ○ at home in a warehouse ○ in a special box ○ at any location

16. Before production, You strictly disinfect and sterilise the pasture and feed, water, and manure channels.

○Never ○Rarely ○Generally ○Often ○Always

17、You use feed additives to promote the growth of cattle, sheep and horses.

○No ○Rarely ○Generally ○Frequently ○Always

18. How strong is your willingness to build sustainable pastures by regularly disinfecting pastures, removing pests, and combating desertification in pastures?

○1 ○2 ○3 ○4 ○5 (1 is the weakest will, 5 is the strongest)

19. Would you like to recycle the manure produced by grazing and the wool shed by cattle and sheep?

○Yes ○No

20、If having to recycle the manure from grazing and collect the shedding wool from cattle and sheep would cause your earnings to drop, how much loss can you afford to incur?

○0-500 yuan ○501-1000 yuan ○1001-1500 yuan ○1501-2000 yuan ○More than 2000 yuan

21. What do you think is the ecological impact of faecal waste, etc., from grazing?

○No impact ○Little impact ○Fair ○Large impact ○Very large impact

22、You will recycle the manure produced by grazing for composting.

○No ○Rarely ○Fairly ○Often ○Always

23. Do you collect dung from grazing as fuel?

○No ○Rarely ○Generally ○Frequently ○Always

24. How strong is your willingness to recycle manure from grazing and wool from cattle and sheep?

○1 ○2 ○3 ○4 ○5 (1 is the weakest will, 5 is the strongest)

Part II (omitted)

Part III (omitted)

Part IV

1. Pasture area (acres):

○ Less than 50 acres ○ 51 acres to 150 acres ○ 151 acres to 250 acres ○ 251 acres or more

2. Number of pasture blocks:

○1~2 ○3~5 ○6~8 ○>8

3. Grassland quality:

○ very poor ○ relatively poor ○ fair ○ relatively good ○ very good

4、Education level:

○First or no knowledge ○Elementary school ○Junior high school

○High school or vocational school ○College and above
5. Number of people in the family livestock labour force:
○1 and below ○2 ○3 ○4 ○5 and above
6. Types of parthenogenesis:
○ Pure animal husbandry ○ Livestock parthenogenesis ○ Work and business
7. Health status of family members:
○Disability ○Major illness ○Chronic illness ○Occasional minor illness ○Healthy
8. Frequency of your participation in livestock breeding skills training:
○Very often ○More often ○Fairly often ○Less often ○Very little often
9. Number of agricultural and livestock machinery owned by the household:
○1 ○2 ○3 ○4 ○5 and above
10. The types of livestock owned by your household are: [multiple choice]
○ Horses ○ Yaks ○Cows ○Sheep ○Goats ○Others
11. The total number of head of livestock sold on your ranch last year was: (head)
○Less than 100 ○100~300 ○300~400 ○Greater than 400
12. Do you own your own home in the city?
○Yes ○No
13. The house in which your family lives:
○ Tent ○ Earth and wood structure ○ Brick and wood structure ○ Reinforced concrete structure
14. Size of your home:
○2 rooms and below ○3-6 rooms ○5-7 rooms ○8 rooms and above
15. Age of your home:
○ Within 5 years ○ 5-10 years ○ 10-20 years ○ 20-30 years ○ 30 years and over
16. Total value of fencing, sheds and machinery owned by your household:
○ Below 10000 ○ 10000~20000 ○ 20000~30000 ○ Above 30000
17. Annual household income ○$20,000 and below:
○$20,001-$40,000 ○$40,001-$60,000 ○$60,000 and above
18. Income from family pastoral farming:
○$10,000 and below ○$10,001~$50,000 ○$50,001~$100,000 ○$100,001 and above
19. Please select the primary source of income for your family
○Wages (income from teachers/enterprises/government departments, etc.)
○Livestock (income from cows/sheep/horses/pigs/chickens/dairy products, etc.)
○Wildlife collection (income from Tibetan medicine/cordyceps, etc.)
○Side business (income from transport/business/crafts/temporary labour, etc.)
○Cultivation (income from barley/oilseed rape/pasture, etc.)
○Others
20. Your family's annual income:
○ very unstable ○ rather unstable ○ average ○ rather stable ○ very stable
21. Does your family have a loan:
○Yes ○No
22. What are the sources of your household's access to credit facilities [Multiple choice]
○bank or credit union ○loan shark ○relative or friend ○other
23. Difficulty in obtaining credit

○ Very difficult ○ Quite difficult ○ Fair ○ Quite easy ○ Very easy

24. Your household's production expenditure [multiple choice]

○Fertilizers/pesticides/agricultural equipment ○Procurement of livestock

○Livestock feed/pharmaceuticals ○ Other

25. Living expenses of your household [Multiple choice]

○Grain/meat/vegetables/non-staple foods ○Clothing and bedding ○New/repaired housing ○Medical care ○Tuition/books Education expenses, etc. ○ Transport expenses ○Other

26. Has your household received subsidies under the grassland ecological conservation grant incentive policy?

○Yes ○No

27. Does anyone in your family have experience as a pastoral committee member?

○Yes ○No
